# A Rare Case of Escitalopram-Induced Acute Dystonia in an Adolescent

**DOI:** 10.1192/j.eurpsy.2026.11128

**Published:** 2026-07-31

**Authors:** T. Pilunthanakul, S. Q. Ting, K. L. C. Ng

**Affiliations:** 1 Ministry of Health Holdings Pte Ltd; 2 Department of Emergency and Crisis Care, Institute of Mental Health, Singapore, Singapore

## Abstract

**Introduction:**

Acute dystonia is a sudden, involuntary contraction of muscles, manifesting as abnormal postures, jaw spasms, or oculogyric crisis. It is most often associated with antipsychotics and certain antiemetics, but rarely with serotonin reuptake inhibitors (SSRIs). Escitalopram is widely prescribed in adolescents due to its favorable safety profile, making dystonia an uncommon and unexpected adverse reaction. In children and adolescents, the presentation may be mistaken for seizures or other neurological conditions, leading to unnecessary investigations and significant distress to families.

**Objectives:**

To describe a rare case of escitalopram-induced acute dystonia in an adolescent following rapid dose escalation, highlight diagnostic challenges, and discuss clinical implications.

**Methods:**

An 18-year-old Indian female with major depressive disorder and social anxiety disorder was started on escitalopram 5mg daily, which was rapidly escalated to 15mg daily within 4 days, and concurrent clonazepam 0.5mg twice daily (BD) as needed (PRN). On the third day, she developed complex dystonic movements, including body alternating between back arching and side-to-side twisting, intermittent bilateral leg flexion and extension, and left-to-right neck twisting, though mainly cocked to one side. However, she remained conscious, was able to follow instructions, was afebrile, and had stable vitals.

**Results:**

A diagnosis of acute dystonia was made, and the patient received intramuscular lorazepam 1mg. Escitalopram was stopped, and clonazepam was switched to lorazepam 0.5mg BD PRN. Symptoms resolved rapidly with no recurrence during observation. Despite reassurance and counselling, the family chose to discharge the patient against medical advice the following day. The antidepressant subsequently prescribed is unknown, and no follow-up information regarding long-term management or recurrence of dystonia was available.

**Conclusions:**

Escitalopram, though generally safe, may precipitate acute dystonia in adolescents, particularly when doses are escalated rapidly. This case underscores the importance of vigilance when initiating or titrating SSRIs in youth. Prompt recognition and timely intervention with benzodiazepines or anticholinergics can prevent prolonged distress and unnecessary investigations. Further pharmacovigilance is needed to clarify risk factors for SSRI-induced extrapyramidal side effects in pediatric populations.

**Disclosure of Interest:**

None Declared

